# Cytokine-Mediated Regulation of Innate Lymphoid Cell Plasticity in Gut Mucosal Immunity

**DOI:** 10.3389/fimmu.2020.585319

**Published:** 2020-12-04

**Authors:** Carlo De Salvo, Kristine-Ann Buela, Theresa T. Pizarro

**Affiliations:** Department of Pathology, Case Western Reserve University School of Medicine, Cleveland, OH, United States

**Keywords:** innate lymphoid cells (ILCs), cytokines, plasticity, gut mucosal immunity, innate immunity

## Abstract

Mucosal barriers are active sites that encounter a bombardment of antigenic stimuli derived from both the commensal flora and a variety of pathogens, as well as from environmental insults. As such, the ability to mount appropriate innate immune responses is an important first line of defense that confers protection to the host. Central to innate immunity are innate lymphoid cells (ILCs), which were first described a decade ago, and represent a family of heterogeneous cells driven by specific transcription factors and exhibit distinct cytokine profiles that are shared with their CD4^+^ T-helper cell counterparts. ILCs are particularly enriched at mucosal surfaces, and the tissue microenvironment and cytokine milieu in which ILCs reside are critical factors that drive the behavior and overall function of these cells. In fact, ILCs situated at mucosal barriers must be able to temper their response to a constant exposure of environmental antigens, but also promptly react to pathogens or signals that are potentially harmful to the host. In this context, the ability of ILCs to readily transdifferentiate in response to their dynamic surroundings has become a vigorous area of research, and defining specific mechanism(s) of ILC plasticity is at the advent of discovery. This review will summarize what is currently known regarding the network of cytokines and regulatory elements that enable ILCs to readily transform, based on the range of diverse signals and signal gradients they encounter that lead to either protective or pathogenic function(s), with focus on the gut mucosal immune system.

## Introduction

Innate lymphoid cells (ILCs) are a diverse family of developmentally-related immune cells that are heterogeneous in their tissue location, cytokine secretion, and effector functions. The term “ILC” has been widely used since 2010, with distinct subsets formally proposed in 2013, based on the transcription factors and specific cytokines regulating their development and function (1). Initially, three groups of ILCs were described, representing innate counterparts that functionally mirror CD4^+^ T-helper cell subsets. More recently, NK and lymphoid tissue inducer (LTi) cells have expanded this family, thereby representing five different ILC groups (2). Group 1 ILCs (ILC1s) express IFNγ and rely on the transcription factor, T-bet, but unlike closely-related NK cells, they do not depend on Eomesodermin (Eomes) and are, in general, non-cytotoxic; however, a phenotypic hybrid of ILC1s and NK cells also exists (3). ILC2s produce the Th2 cytokines, IL-4, IL-5, and IL-13, as well as IL-9 and amphiregulin, and are functionally influenced by the transcription factors, GATA3 and RORα. ILC3s utilize RORγt to drive production of IL-22, but also IL-17, and are distinguished into further subsets based on expression of the natural cytotoxicity receptors (NCRs), NKp46 and NKp44. LTi cells are also dependent on RORγt, but produce lymphotoxin and are critical for secondary lymphoid organ formation, including Peyer’s patches. Although organizing ILCs into these five subgroups provides a basic infrastructure to understand the ILC family in regards to development and function, it is also important to consider whether this diversity is discrete, or reflects a given subset’s adaptability to the changing tissue microenvironment, such as what occurs during disease pathogenesis, and for these cells to undergo transformation.

In this context, several significant questions remain unanswered regarding the mechanisms underlying ILC development, divergence, and differentiation. What kind of environmental cues and other regulatory factors are necessary to determine cell fate? How do these processes relate to that of the CD4^+^ T cell helper population? Which ILC subgroups are terminally-differentiated, or like their corresponding CD4^+^ T helper cells, can convert from one ILC type to another? Although investigation of ILC plasticity is still in its infancy, these questions are now being answered, particularly with the emergence of more advanced techniques, such as single-cell transcriptomics, which facilitates a better understanding of the complexity and heterogeneity of ILCs (4–7).

In general, ILC2s and ILC3s have the ability to transdifferentiate into ILC1s, which is a reversible process, and highly dependent on the cytokine milieu and tissue microenvironment in which these cells reside. Transformation of ILC2s to ILC3s has also been reported, while plasticity between “natural” and “inflammatory” ILC2s, as well as of NK cells to ILC1s, may represent transient stages or terminally-differentiated events during disease pathogenesis, respectively. The following sections will summarize current findings regarding ILC plasticity and what signals (*e.g*., cytokines) control these processes, with particular focus on the gut mucosal immune system. We will also discuss the more recently coined regulatory ILC (ILCreg) subset and its contribution to mucosal immunity.

## Cytokines that Regulate Plasticity of LTi and ILC3s

Plasticity within ILC subgroups was first observed in LTi cells, in which a gradient of RORγt expression is stabilized by IL-7 and the gut microbiome, while IL-12 and IL-15 accelerate its loss. Specifically, RORγt^+^ LTi cells produce IL-22 and are functionally protective, whereas RORγt^−^ LTi cells secrete IFNγ and can induce colitis (8, 9). Similarly, ILC3s that are NKp46^-^CCR6^-/low^ are able to differentiate into NKp46^+^ ILC3s, depending on upregulation of the prototypic ILC1 transcription factor, T-bet, which stimulates IFNγ and IL-22 production that are important for protection against *Salmonella* infection (10). In addition, Notch signaling mediated by T-bet guides the development of NKp46^+^ ILC3s (11). Interestingly, this upregulation of T-bet in a subpopulation of ILC3s is concurrent with loss of RORγt expression (8, 12).

How these “ex-ILC3s” are generated is of great interest and the focus of recent investigation. Although different mechanisms have been implicated, the tissue-specific microenvironment appears to be essential to skew ILC3 identity, as the frequency of these subsets differs depending on their residence. Most abundant within the intestines are ILC3s, wherein CCR6^+^ ILC3s and/or LTi-like cells are most prominently found in cryptopatches, whereas NKp46^+/-^CCR6^-/low^ ILC3s are located within the lamina propria. Interestingly, Pearson et al., recently demonstrated that ILC3s can mobilize from intestinal cryptopatches to the lamina propria, and this migration is dependent on GM-CSF (13). Another mechanism for ILC3 switching was revealed through the use of RORγt-GFP reporter mice (14), which have been instrumental for lineage-tracing experiments (15); these mice retain GFP expression in their RORγt^+^ cells, even after loss of RORγt expression. Studies show that within the ILC1 subset, some cells have the ability to produce IFNγ, a typical ILC1 cytokine, and still have traceable GFP expression, suggesting that they were likely once RORγt^+^ ILC3s (*i.e*., ex-ILC3s) (8–10, 16–18). Although ILC3s rely on IL-7 signaling for proper development and maintenance, ex-ILC3s downregulate CD127 and c-Kit, and are more responsive to IL-15; in contrast, all other ILC3 subsets are not entirely dependent on IL-15 signaling (18, 19). Furthermore, upon IL-23 stimulation, STAT4/T-bet-dependent regulation of NCR^+^ ILC3s can promote IFNγ production and plasticity towards type 1 fate (20).

More recently, an intermediate population has been identified in human tonsils and small intestines, with characteristics of both ILC3s and intraepithelial CD103^+^ ILC1s (21). This population expresses CD103, CCR6, and CD300LF to different degrees, with scRNA-seq analysis providing evidence that ILC3s are able to convert *in vivo* into CD103^+^ ILC1s. Furthermore, Aiolos (encoded by *IKZF3*), a member of the Ikaros family of transcription factors, and expressed mainly by ILC1s and NK cells, is critical for skewing ILC3s into CD103^-^ (22) and intraepithelial CD103^+^ ILC1s (21). The transcription factor, c-Maf, is also implicated in controlling the homeostatic balance of ILC3s by directly inhibiting T-bet, and therefore transdifferentiation to ILC1s (23, 24); in the absence of c-Maf, CD196^-^ ILC3s transdifferentiate to an ILC1 phenotype (23). Another factor, BCL6, regulates ILC3-to-ILC1 plasticity by repressing ILC3-promoting pathways, such as IL-23-induced signaling (24), resulting in reduced ILC1 frequency.

In humans, the transition from ILC3s to ILC1s is contingent on downstream signals directed by IL-12 that induce the expression of T-bet (12). This is the case in inflamed intestinal tissues from Crohn’s disease patients, wherein increased ILC1s, at the expense of ILC3s, is observed, highlighting the prevalence of ILC3s during homeostasis, possibly by supporting T regulatory cell (Treg) activity *via* IL-2 (25), and ILC1s during (pathogenic) inflammatory events (26). Interestingly, differentiation from NKp46^-^ to NKp46^+^ ILC3s, and then to NKp46^+^ ex-ILC3s (*i.e*., “ILC1s”), modulated by low-to-high T-bet expression, is reversible both *in vivo* and *in vitro*, and is dependent on IL-23, IL-2, and IL-1β, and is further enhanced by retinoic acid (12). This ILC3-to-ILC1 polarization also depends on the presence of CD14^+^ and CD14^-^ dendritic cells, wherein an increase in the former promotes ILC1 differentiation, and an increase in the latter, induces ILC3 skewing. These findings indicate that, as much as the cytokine milieu and activation of transcription factors can affect ILC subset composition, so can environmental signals from local immune cells. In other studies, fate-mapping experiments have established that NKp46^+^RORγt^+^ ILC3s can downregulate *in vivo* expression of NKp46, generating NKp46^-^RORγt^+^ ILC3s (11, 27).

As mentioned earlier, T-bet itself can direct the development of NKp46^+^ ILC3s, which is mediated through Notch signaling (10). Confounding this finding, however, Notch is also reported to regulate plasticity within ILC3 subsets by controlling the fate of NKp46^+^ ILC3s (28). Furthermore, interconversion within the ILC3 subset occurs in response to TGFβ (29). Transcripts for the two subunits of the TGFβ receptor, TGFβ receptor I and II, can be detected in ILCs (30). Deletion of TGFβ receptor II leads to expansion of NKp46^+^ ILC3s, indicating that TGFβ impairs the development of NKp46^+^ ILC3s. Additionally, TGFβ antagonizes Notch signaling, implying that the ILC3 phenotype depends on fine-tuning the divergent effects of both TGFβ and Notch signaling that may be required to preserve homeostasis, *in vivo* (29).

**Figure 1:**
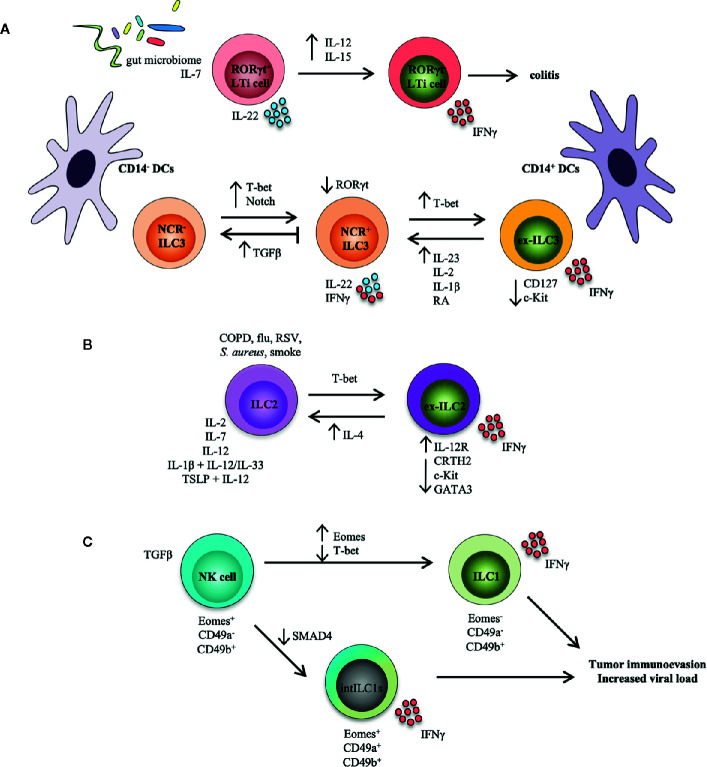
ILC transdifferentiation occurs in response to cytokine and/or pathogen stimulation. Schematic representation summarizing plasticity among ILC populations reported both in mouse models and in human settings. **(A)** Plasticity within the LTi and ILC3 populations. RORγt in LTi cells is stabilized by IL-7 and the gut microbiome; these cells produce IL-22 and are functionally protective. Conversely, IL-12 and IL-15 accelerate loss of RORγt in LTi cells, which secrete IFNγ and induce colitis. NCR^-^ ILC3s can convert into NCR^+^ ILC3s under the influence of T-bet and Notch. T-bet upregulation of NCR^+^ ILC3s can transform ILC3s into ILC1-like cells (*i.e*., ex-ILC3s) that downregulate expression of CD127 and c-Kit, and secrete IFNγ. This process is reversed by IL-23, IL-1β, IL-2, and retinoic acid (RA), while TGFβ inhibits NCR^+^ ILC3 development from NCR^-^ ILC3s and possibly drives reversion of these cells. Increased presence of CD14^+^ dendritic cells (DCs) drives an ex-ILC3 phenotype, while CD14^-^ DCs promote the generation of NCR^-^ ILC3s. **(B)** Plasticity within the ILC2 population. In the presence of an inflammatory environment typical of COPD (chronic obstructive pulmonary disease), smoke, and bacterial or viral infection, or after stimulation with IL-2, IL-7, IL-12, IL-1β, and IL-12, as well as TSLP and IL-12, ILC2s can assume an ILC1-like phenotype (*i.e*., ex-ILC2) that upregulates expression of the IL-12 receptor, downregulates ILC2-associated proteins (CRTH2, c-Kit, GATA3), and produces IFNγ. GATA3, a critical transcription factor for maintaining ILC2 identity, is repressed by T-bet. This route can also be reversed by eosinophil-derived IL-4. **(C)** Plasticity within the NK and ILC1 populations. In the context of a tumor microenvironment, NK cells (Eomes^+^CD49a^-^CD49b^+^) can convert to intermediate ILC1s (Eomes^+^CD49a^+^CD49b^+^), or intILC1s, upon stimulation with TGFβ. Similarly, NK cells can also transform to real ILC1s (Eomes^-^CD49a^-^CD49b^+^). SMAD4 promotes TGFβ signaling, but the lack of SMAD4 in NK cells transforms them into intILC1s. These ex-NK cells are not functional however, and provide a mechanism for tumors to escape surveillance, and may allow for increased viral burden in infection.

Taken together, these studies indicate that ILC3s exhibit bi-directional differentiation that can be modulated by T-bet and RORγt gradients within the ILC3 lineage (**Figure 1A**). Are ex-ILC3s generated from NCR^+^ ILC3s, and are they all dependent on Notch and TGF-β signaling? How does T-bet and RORγt detect the extent of inflammation and deliver equivalent immune responses? To determine how these transcription factors readily impose various ILC3 effector programs, it is imperative to recognize the underlying molecular mechanisms for ILC3 plasticity. How this balance is maintained could reflect blunted inflammatory responses (*i.e*., in presence of commensal flora during homeostasis), while being poised to mount a vigorous immune reaction when challenge or insult occurs, particularly at mucosal barriers. Various ILC3 subsets may be actively modulated by the temporal degree of inflammation that then directs T-bet and RORγt expression.

## Cytokines that Regulate Plasticity of ILC2s

ILC2s have also been reported to alter their functional and transcriptional programs. ILC2s are capable of converting to IFNγ-producing ILC1s in both mice and humans (31–33). Specifically, ILC2s derived from human blood proliferate *in vitro* in the presence of IL-2 and IL-7, upregulate T-bet, and secrete IFNγ *via* IL-12 (34). Other studies show that, in the presence of IL-1β, human ILC2s not only express T-bet, but also the IL-12 receptor subunits, IL-12RB1 and IL-12RB2, enabling ILC2s to respond to IL-12 (32). Interestingly, patients with defects in IL-12Rβ1 suffer from a syndrome referred to as Mendelian susceptibility to mycobacterial disease; these individuals are not capable of generating ILC2s that potentially can convert to ILC1s (34). IL-12 is also important in inducing genomic modifications in the *IFNγ* locus, allowing for IFNγ secretion; these IFNγ-expressing “ex-ILC2s” are also able to secrete IL-13 (32). Similarly, Bal et al. showed in an inflammatory environment (*i.e*., lung tissues of patients with chronic obstructive pulmonary disease) that ILC2s can convert into IFNγ-producing ILC1s by exposure to either combination IL-1β and IL-12, IL-33, or TSLP (thymic stromal lymphopoietin) and IL-12 (31). After adoptive transfer into humanized mice, these ILC2s downregulate chemoattractant receptor-homologous molecule expressed on T-helper type 2 cells (CRTH2) and c-Kit expression, which are typical markers for ILC2s (31). These CRTH2^-^Kit^-^ ex-ILC2s also express increased Tbx21 (T-bet) compared to CRTH2^+^Kit^+^ ILC2s. In humans, two subsets of CRTH2^+^ ILC2s were identified in peripheral blood, based on CD117 expression: CD117^-^ ILC2s, indicating mature ILC2s, and CD117^+^ ILC2s, showing some features of ILC3s, such as RORγt expression (35, 36). Upon IL-33 stimulation, CD117^+^ ILC2s produce Th2 cytokines, whereas IL1β and IL-23 induce these cells to produce IL-17 (35, 36) and CCR6, which is also expressed by IL-17-producing ILC3s (37). ILC2s have also been reported to display phenotypic plasticity in response to infectious agents, such as influenza virus, respiratory syncytial virus, *Staphylococcus aureus*, and interestingly, cigarette smoke (33). GATA3 expression is downregulated in ILC2s in response to exposure to these agents, with a subsequent increase in IL-12Rβ2, IL-18Rα, and T-bet. This effect was confirmed *in vivo* by adoptive transfer of ILC2s from ST2/IL-33R reporter mice into recipients lacking T cells and ILCs (*Rag2* ^-/-^ *Il2rg* ^-/-^ mice) that were then infected with influenza. Although donor ILC2s downregulate GATA3 and upregulate IL-18Rα and IL-12Rβ2, they do not express T-bet; however, upon stimulation with IL-12 and IL-18, a portion of these ex-ILC2s are capable of IFNγ secretion, suggesting skewing towards ILC1s (33).

Interestingly, similar to ILC3-to-ILC1 conversion, ILC2-to-ILC1 transdifferentiation is also reversible. Although the mechanism for this process is not entirely clear, eosinophil-derived IL-4 appears to prevent IL-12-mediated ILC2 differentiation to ILC1 in an inflammatory milieu, such as within nasal polyps of patients with chronic rhinosinusitis (31). Furthermore, expression of the receptors for IL-1β, IL-12, IL-18, and IL-33 influence ILC2 expansion and T-bet induction, facilitating ILC2 conversion towards an ILC1 phenotype (31–33).

TGFβ from pulmonary epithelial cells has been observed to promote allergic immune responses by expanding IL-13–secreting ILC2s (30). IL-33 induces proliferation of ILC2s and stimulates TGFβ secretion in lung airways to activate ILC2 function and migration (30). The possibility therefore exists that TGFβ, in line with other inflammatory mediators, acts as a modulator of ILC2 and ILC3 fate. In fact, it has been proposed that murine ILC2s can be categorized into two subsets: a transient “inflammatory” subpopulation whose fate and function is dependent on the transcription factor, BATF, and expresses more IL-25R (thereby responding preferentially to IL-25), and a tissue resident “natural” ST2^+^ ILC2 subpopulation induced by IL-33 (38, 39). Inflammatory (i)ILC2s are not responsive to IL-33, but can convert into natural (n)ILC2s, both *in vivo* and *in vitro*, and express low quantities of RORγt and upon stimulation, IL-17 and IL-13, indicating plasticity between iILC2s and ILC3-like cells (38).

Although this phenomenon has not been recapitulated in humans, human clones of ILC2s and ILC3s that secrete both IL-22 and IL-13 have been characterized (40, 41). Similarly, ILC2s can respond to inflammation in skin and lungs by transdifferentiating into IL-17-producing ILC3s; specifically, ILC2s co-cultured with dermal cells and the fungus, *Candida albicans*, produce IL-17 and acquire a phenotype similar to that of NKp44^-^ ILC3s (35). Finally, patients with more severe non-allergic asthma, with blunted Th2 responses, display both IL-5- and IL-13-producing ILC2s, and IL-17-producing ILC3s and ILC2s (42).

Together, these studies provide support for the existence of ILC2 phenotypic flexibility (**Figure 1B**). Future studies will benefit from experiments that can trace genetic lineages to clarify issues related to ILC2 plasticity. Discriminating between *in vivo* expansion of already low numbers of ILC subsets vs. transdifferentiation is challenging. Identifying extracellular influences and why lineage flexibility is necessary, will facilitate knowledge as to how pathogenic responses can be controlled and skewed toward protective function(s), leading to potential therapeutic interventions (8, 43).

## Cytokines That Regulate Plasticity of ILC1s and NK Cells

Previous sections of this review have highlighted interconversion of ILC3s and ILC2s into ILC1s, and vice-versa. This section will examine ILC plasticity of the closely-related subgroups, NK cells and ILC1s. Although both subsets possess identical cell surface markers (NKp46, NKG2D), parallel transcriptional programming, and similar cytokine profiles (IFNγ, TNF), they are now considered distinct populations. For instance, NK cells developmentally do not need GATA3 (44), but some ILC1s do (45–48). The proposed cellular basis of this divergence is due to the transcription factors, inhibitor of DNA binding protein-2 (ID2) and promyelocytic leukemia zinc finger protein (PLZF), which are expressed in ILC precursors, but not in NK cells (18, 49). Recent studies, however, using polychromic reporter mice, show that ILC precursors actually have considerable NK precursor activity, challenging the existing paradigm that considers ILC1 and NK cells as two different subsets (50, 51). Moreover, conversion of NK cells into ILC1s is observed in mice with non-alcoholic fatty liver disease, and likely mediated, in part, by TGFβ (52). NK cells also express Eomes that, together with T-bet, modulate production of lysis-associated granules containing perforin and granzyme (53). Therefore, conceptually, if ILCs are innate counterparts of CD4^+^ T helper cells, then NK cells represent the innate version of CD8^+^ T cells.

Current investigation, however, suggests that mature NK cells and ILC1s can also undergo plasticity between themselves. Forced Eomes expression in T-bet^+^ ILC1s is, in fact, sufficient for transformation to CD49b^+^ NK cells (54). Conversely, IL-12 can convert Eomes^+^ NK cells into Eomes^-^ ILC1-like cells following infection with *Toxoplasma gondii* (55). Intriguingly, in humans, an Eomes^+^Tbet^lo^ liver-resident NK cell subset has been identified (56, 57). More recently, two reports show the requirement for TGFβ in driving NK cell-to-ILC1 conversion in a tumor microenvironment (58, 59) and during virus infection (59). Gao et al., recently identified NK cells that convert into intermediate ILC1s (intILC1s), also *via* TGFβ (58). Surprisingly, even though it is well-established that SMAD4 promotes signaling by TGFβ family members, and TGFβ encourages skewing towards the ILC1 lineage, Cortez, et al. demonstrated that SMAD4 deficiency in ILC1s does not affect their differentiation, but instead converts SMAD4-deficient NK cells into ILC1-like cells (59). This suggests that SMAD4 acts as a negative regulator of TGFβ signaling in NK cells, and inhibits typical TGFβ imprinting that is characteristic of ILC1s. More importantly, these intILC1s possess gene expression profiles that are intermediate between ILC1s and NK cells, and are incapable of limiting tumor burden or viral load. Whether these ILC1-like cells can actually revert back to NK cells is uncertain, but these studies reveal a mechanism by which the ILC continuum is controlled by a rheostat that includes a cytokine milieu driven by the local tissue microenvironment (**Figure 1C**).

## New ILC on the Block: ILCregs

Like Tregs, ILCs are reported to have a corresponding population, aptly-called ILCregs (60). Wang, et al. identified ILCregs in mouse and human intestines that are induced upon inflammatory stimuli, such as DSS, anti-CD40 antibody, as well as *Salmonella typhimurium* and *Citrobacter rodentium* infection (60). ILCregs purportedly originate from common helper ILC precursors (CHILP), and not ILC precursors, and express the transcription factors, ID3 and Sox4. Although ILCregs do not express CD4 or Foxp3, they possess a gene identity distinct from other ILC subsets and Tregs. Importantly, ILCregs produce IL-10 and TGFβ, and suppress activation of ILC1s and ILC3s, but not ILC2s, in an IL-10-dependent manner. TGFβ is necessary for maintenance of ILCregs and autocrine TGFβ is required for its expansion during intestinal inflammation. The role of ILCregs in colorectal cancer (CRC) show that these cells transdifferentiate from ILC3s during CRC progression *via* TGFβ, indicating potential pro-tumorigenic function during ILC3-to-ILCreg plasticity (61). Furthermore, retinoic acid is reported to induce transdifferentiation of ILC2s into IL-10-producing ILCregs during airway inflammation (62), while ILC2s provide a predominant and inducible source of IL-10 in the GI tract (63). The existence of this novel ILC subset is, however, controversial and the topic of current investigation that elicits several open-ended questions. How do the transcription factors, ID3 and Sox4, synergize to modulate ILCreg development? Since ILCregs constitutively express *Tgfbr1, Tgfbr2, Il2rb*, and *Il2rg*, can they be stimulated directly by IL-2 and TGFβ? Why are ILC2s unresponsive to the effects of IL-10-producing ILCregs? It is interesting to note that a subset of IL-10-secreting ILC2s, detected in murine lungs after IL-33 treatment or papain stimulation, associates with reduced lung eosinophilia (64). Comparable to ILCregs, these IL-10^+^ ILC2s also express the anti-inflammatory gene, *Retnla*, thereby suggesting anti-inflammatory properties, yet warrants further investigation in other pathogenic inflammatory states.

## Conclusions and Future Directions

ILCs are particularly enriched at mucosal surfaces, and studies over the last decade highlight various functions that are important at the intestinal barrier (65), both in maintaining gut homeostasis, but also during chronic inflammation. ILC plasticity allows adaptability in response to changes in the local tissue microenvironment that are critical to appropriately respond to pathogenic challenge, without the need for *de novo* ILC generation and recruitment. Investigation into the precise mechanisms that control plasticity of specific ILC subsets, particularly at mucosal surfaces, is in its infancy and will aid in further understanding disease pathogenesis and designing targeted therapies in the future.

## Author Contributions

CS and K-AB contributed equally to researching, writing of initial drafts, and assembling manuscript. TP conceptualized, edited, and assembled the final submitted manuscript. All authors contributed to the article and approved the submitted version.

## Funding

This work was supported by grants from the National Institutes of Health: DK091222, DK042191 (*TTP*), and DK097948 Pilot and Feasibility Award (*CDS*); and the Crohn’s & Colitis Foundation: CDA-581292 (*CDS*) and RFA-410354 (*KGB*).

## Conflict of Interest

The authors declare that the research was conducted in the absence of any commercial or financial relationships that could be construed as a potential conflict of interest.

## Abbreviations

c-Maf, musculoaponeurotic fibrosarcoma oncogene homolog; Eomes, Eomesodermin; GATA3, GATA binding protein-3; GFP, green fluorescent protein; GM-CSF, granulocyte macrophage colony stimulating factor; IFNγ, interferon-γ; IL-, interleukin-; ILC, innate lymphoid cell; ILCreg, regulatory innate lymphoid cell; LTi, lymphoid tissue inducer; NCR, natural cytotoxicity receptor; NK, natural killer; TGFβ, transforming growth factor-beta; TSLP, thymic stromal lymphopoietin; Th, T-helper; Treg, T-regulatory cell.

## References

[1] Spits H Artis D Colonna M Diefenbach A Di Santo JP Eberl G Innate lymphoid cells–a proposal for uniform nomenclature. Nat Rev Immunol (2013) 13(2):145–9. 10.1038/nri3365 23348417

[2] Vivier E Artis D Colonna M Diefenbach A Di Santo JP Eberl G Innate Lymphoid Cells: 10 Years On. Cell (2018) 174(5):1054–66. 10.1016/j.cell.2018.07.017 30142344

[3] Spits H Bernink JH Lanier L NK cells and type 1 innate lymphoid cells: partners in host defense. Nat Immunol (2016) 17(7):758–64. 10.1038/ni.3482 27328005

[4] Gury-BenAri M Thaiss CA Serafini N Winter DR Giladi A Lara-Astiaso D The Spectrum and Regulatory Landscape of Intestinal Innate Lymphoid Cells Are Shaped by the Microbiome. Cell (2016) 166(5):1231–46.e13. 10.1016/j.cell.2016.07.043 27545347

[5] Yu Y Tsang JC Wang C Clare S Wang J Chen X Single-cell RNA-seq identifies a PD-1hi ILC progenitor and defines its development pathway. Nature (2016) 539(7627):102–6. 10.1038/nature20105 27749818

[6] Ishizuka IE Chea S Gudjonson H Constantinides MG Dinner AR Bendelac A Single-cell analysis defines the divergence between the innate lymphoid cell lineage and lymphoid tissue-inducer cell lineage. Nat Immunol (2016) 17(3):269–76. 10.1038/ni.3344 PMC4755916 26779601

[7] Bjorklund AK Forkel M Picelli S Konya V Theorell J Friberg D The heterogeneity of human CD127(+) innate lymphoid cells revealed by single-cell RNA sequencing. Nat Immunol (2016) 17(4):451–60. 10.1038/ni.3368 26878113

[8] Vonarbourg C Mortha A Bui VL Hernandez PP Kiss EA Hoyler T Regulated expression of nuclear receptor RORgammat confers distinct functional fates to NK cell receptor-expressing RORgammat(+) innate lymphocytes. Immunity (2010) 33(5):736–51. 10.1016/j.immuni.2010.10.017 PMC3042726 21093318

[9] Cella M Otero K Colonna M Expansion of human NK-22 cells with IL-7, IL-2, and IL-1beta reveals intrinsic functional plasticity. Proc Natl Acad Sci U S A (2010) 107(24):10961–6. 10.1073/pnas.1005641107 PMC2890739 20534450

[10] Klose CS Kiss EA Schwierzeck V Ebert K Hoyler T d’Hargues Y A T-bet gradient controls the fate and function of CCR6-RORgammat+ innate lymphoid cells. Nature (2013) 494(7436):261–5. 10.1038/nature11813 23334414

[11] Rankin LC Groom JR Chopin M Herold MJ Walker JA Mielke LA The transcription factor T-bet is essential for the development of NKp46+ innate lymphocytes via the Notch pathway. Nat Immunol (2013) 14(4):389–95. 10.1038/ni.2545 PMC4076532 23455676

[12] Bernink JH Krabbendam L Germar K de Jong E Gronke K Kofoed-Nielsen M Interleukin-12 and -23 Control Plasticity of CD127(+) Group 1 and Group 3 Innate Lymphoid Cells in the Intestinal Lamina Propria. Immunity (2015) 43(1):146–60. 10.1016/j.immuni.2015.06.019 26187413

[13] Pearson C Thornton EE McKenzie B Schaupp AL Huskens N Griseri T ILC3 GM-CSF production and mobilisation orchestrate acute intestinal inflammation. Elife (2016) 5:e10066. 10.7554/eLife.10066 26780670 PMC4733039

[14] Eberl G Marmon S Sunshine MJ Rennert PD Choi Y Littman DR An essential function for the nuclear receptor RORgamma(t) in the generation of fetal lymphoid tissue inducer cells. Nat Immunol (2004) 5(1):64–73. 10.1038/ni1022 14691482

[15] Sawa S Cherrier M Lochner M Satoh-Takayama N Fehling HJ Langa F Lineage relationship analysis of RORgammat+ innate lymphoid cells. Science (2010) 330(6004):665–9. 10.1126/science.1194597 20929731

[16] Fuchs A Vermi W Lee JS Lonardi S Gilfillan S Newberry RD Intraepithelial type 1 innate lymphoid cells are a unique subset of IL-12- and IL-15-responsive IFN-gamma-producing cells. Immunity (2013) 38(4):769–81. 10.1016/j.immuni.2013.02.010 PMC3634355 23453631

[17] Bernink JH Peters CP Munneke M te Velde AA Meijer SL Weijer K Human type 1 innate lymphoid cells accumulate in inflamed mucosal tissues. Nat Immunol (2013) 14(3):221–9. 10.1038/ni.2534 23334791

[18] Klose CS Flach M Mohle L Rogell L Hoyler T Ebert K Differentiation of type 1 ILCs from a common progenitor to all helper-like innate lymphoid cell lineages. Cell (2014) 157(2):340–56. 10.1016/j.cell.2014.03.030 24725403

[19] Satoh-Takayama N Lesjean-Pottier S Vieira P Sawa S Eberl G Vosshenrich CA IL-7 and IL-15 independently program the differentiation of intestinal CD3-NKp46+ cell subsets from Id2-dependent precursors. J Exp Med (2010) 207(2):273–80. 10.1084/jem.20092029 PMC2822619 20142427

[20] Mikami Y Scarno G Zitti B Shih HY Kanno Y Santoni A NCR(+) ILC3 maintain larger STAT4 reservoir via T-BET to regulate type 1 features upon IL-23 stimulation in mice. Eur J Immunol (2018) 48(7):1174–80. 10.1002/eji.201847480 PMC11170443 29524223

[21] Cella M Gamini R Secca C Collins PL Zhao S Peng V Subsets of ILC3-ILC1-like cells generate a diversity spectrum of innate lymphoid cells in human mucosal tissues. Nat Immunol (2019) 20(8):980–91. 10.1038/s41590-019-0425-y PMC6685551 31209406

[22] Mazzurana L Forkel M Rao A Van Acker A Kokkinou E Ichiya T Suppression of Aiolos and Ikaros expression by lenalidomide reduces human ILC3-ILC1/NK cell transdifferentiation. Eur J Immunol (2019) 49(9):1344–55. 10.1002/eji.201848075 31151137

[23] Parker ME Barrera A Wheaton JD Zuberbuehler MK Allan DSJ Carlyle JR c-Maf regulates the plasticity of group 3 innate lymphoid cells by restraining the type 1 program. J Exp Med (2020) 217(1). 10.1084/jem.20191030 PMC7037249 31570496

[24] Pokrovskii M Hall JA Ochayon DE Yi R Chaimowitz NS Seelamneni H Characterization of Transcriptional Regulatory Networks that Promote and Restrict Identities and Functions of Intestinal Innate Lymphoid Cells. Immunity (2019) 51(1):185–97.e6. 10.1016/j.immuni.2019.06.001 31278058 PMC6863506

[25] Zhou L Chu C Teng F Bessman NJ Goc J Santosa EK Innate lymphoid cells support regulatory T cells in the intestine through interleukin-2. Nature (2019) 568(7752):405–9. 10.1038/s41586-019-1082-x PMC6481643 30944470

[26] Bal SM Golebski K Spits H Plasticity of innate lymphoid cell subsets. Nat Rev Immunol (2020). 10.1038/s41577-020-0282-9 32107466

[27] Rankin LC Girard-Madoux MJ Seillet C Mielke LA Kerdiles Y Fenis A Complementarity and redundancy of IL-22-producing innate lymphoid cells. Nat Immunol (2016) 17(2):179–86. 10.1038/ni.3332 PMC4720992 26595889

[28] Chea S Perchet T Petit M Verrier T Guy-Grand D Banchi EG Notch signaling in group 3 innate lymphoid cells modulates their plasticity. Sci Signaling (2016) 9(426):ra45. 10.1126/scisignal.aaf2223 27141929

[29] Viant C Rankin LC Girard-Madoux MJH Seillet C Shi W Smyth MJ Transforming growth factor-beta and Notch ligands act as opposing environmental cues in regulating the plasticity of type 3 innate lymphoid cells. Sci Signaling (2016) 9(426):ra46. 10.1126/scisignal.aaf2176 27141930

[30] Denney L Byrne AJ Shea TJ Buckley JS Pease JE Herledan GM Pulmonary Epithelial Cell-Derived Cytokine TGF-beta1 Is a Critical Cofactor for Enhanced Innate Lymphoid Cell Function. Immunity (2015) 43(5):945–58. 10.1016/j.immuni.2015.10.012 PMC4658339 26588780

[31] Bal SM Bernink JH Nagasawa M Groot J Shikhagaie MM Golebski K IL-1beta, IL-4 and IL-12 control the fate of group 2 innate lymphoid cells in human airway inflammation in the lungs. Nat Immunol (2016) 17(6):636–45. 10.1038/ni.3444 27111145

[32] Ohne Y Silver JS Thompson-Snipes L Collet MA Blanck JP Cantarel BL IL-1 is a critical regulator of group 2 innate lymphoid cell function and plasticity. Nat Immunol (2016) 17(6):646–55. 10.1038/ni.3447 27111142

[33] Silver JS Kearley J Copenhaver AM Sanden C Mori M Yu L Inflammatory triggers associated with exacerbations of COPD orchestrate plasticity of group 2 innate lymphoid cells in the lungs. Nat Immunol (2016) 17(6):626–35. 10.1038/ni.3443 PMC5345745 27111143

[34] Lim AI Menegatti S Bustamante J Le Bourhis L Allez M Rogge L IL-12 drives functional plasticity of human group 2 innate lymphoid cells. J Exp Med (2016) 213(4):569–83. 10.1084/jem.20151750 PMC4821648 26976630

[35] Bernink JH Ohne Y Teunissen MBM Wang J Wu J Krabbendam L c-Kit-positive ILC2s exhibit an ILC3-like signature that may contribute to IL-17-mediated pathologies. Nat Immunol (2019) 20(8):992–1003. 10.1038/s41590-019-0423-0 31263279

[36] Hochdorfer T Winkler C Pardali K Mjosberg J Expression of c-Kit discriminates between two functionally distinct subsets of human type 2 innate lymphoid cells. Eur J Immunol (2019) 49(6):884–93. 10.1002/eji.201848006 30892687

[37] Golebski K Ros XR Nagasawa M van Tol S Heesters BA Aglmous H IL-1beta, IL-23, and TGF-beta drive plasticity of human ILC2s towards IL-17-producing ILCs in nasal inflammation. Nat Commun (2019) 10(1):2162. 10.1038/s41467-019-09883-7 31089134 PMC6517442

[38] Huang Y Guo L Qiu J Chen X Hu-Li J Siebenlist U IL-25-responsive, lineage-negative KLRG1(hi) cells are multipotential ‘inflammatory’ type 2 innate lymphoid cells. Nat Immunol (2015) 16(2):161–9. 10.1038/ni.3078 PMC4297567 25531830

[39] Miller MM Patel PS Bao K Danhorn T O’Connor BP Reinhardt RL BATF acts as an essential regulator of IL-25-responsive migratory ILC2 cell fate and function. Sci Immunol (2020) 5(43). 10.1126/sciimmunol.aay3994 PMC7112987 31924686

[40] Mjosberg JM Trifari S Crellin NK Peters CP van Drunen CM Piet B Human IL-25- and IL-33-responsive type 2 innate lymphoid cells are defined by expression of CRTH2 and CD161. Nat Immunol (2011) 12(11):1055–62. 10.1038/ni.2104 21909091

[41] Crellin NK Trifari S Kaplan CD Satoh-Takayama N Di Santo JP Spits H Regulation of cytokine secretion in human CD127(+) LTi-like innate lymphoid cells by Toll-like receptor 2. Immunity (2010) 33(5):752–64. 10.1016/j.immuni.2010.10.012 21055975

[42] Cai T Qiu J Ji Y Li W Ding Z Suo C IL-17-producing ST2(+) group 2 innate lymphoid cells play a pathogenic role in lung inflammation. J Allergy Clin Immunol (2019) 143(1):229–44.e9. 10.1016/j.jaci.2018.03.007 29625134 PMC6170730

[43] Buonocore S Ahern PP Uhlig HH Ivanov II Littman DR Maloy KJ Innate lymphoid cells drive interleukin-23-dependent innate intestinal pathology. Nature (2010) 464(7293):1371–5. 10.1038/nature08949 PMC3796764 20393462

[44] Samson SI Richard O Tavian M Ranson T Vosshenrich CA Colucci F GATA-3 promotes maturation, IFN-gamma production, and liver-specific homing of NK cells. Immunity (2003) 19(5):701–11. 10.1016/S1074-7613(03)00294-2 14614857

[45] Hoyler T Klose CS Souabni A Turqueti-Neves A Pfeifer D Rawlins EL The transcription factor GATA-3 controls cell fate and maintenance of type 2 innate lymphoid cells. Immunity (2012) 37(4):634–48. 10.1016/j.immuni.2012.06.020 PMC3662874 23063333

[46] Yagi R Zhong C Northrup DL Yu F Bouladoux N Spencer S The transcription factor GATA3 is critical for the development of all IL-7Ralpha-expressing innate lymphoid cells. Immunity (2014) 40(3):378–88. 10.1016/j.immuni.2014.01.012 PMC4026797 24631153

[47] Klein Wolterink RG Serafini N van Nimwegen M Vosshenrich CA de Bruijn MJ Fonseca Pereira D Essential, dose-dependent role for the transcription factor Gata3 in the development of IL-5+ and IL-13+ type 2 innate lymphoid cells. Proc Natl Acad Sci U S A (2013) 110(25):10240–5. 10.1073/pnas.1217158110 PMC3690884 23733962

[48] Serafini N Klein Wolterink RG Satoh-Takayama N Xu W Vosshenrich CA Hendriks RW Gata3 drives development of RORgammat+ group 3 innate lymphoid cells. J Exp Med (2014) 211(2):199–208. 10.1084/jem.20131038 24419270 PMC3920560

[49] Constantinides MG McDonald BD Verhoef PA Bendelac A A committed precursor to innate lymphoid cells. Nature (2014) 508(7496):397–401. 10.1038/nature13047 24509713 PMC4003507

[50] Xu W Cherrier DE Chea S Vosshenrich C Serafini N Petit M An Id2(RFP)-Reporter Mouse Redefines Innate Lymphoid Cell Precursor Potentials. Immunity (2019) 50(4):1054–68.e3. 10.1016/j.immuni.2019.02.022 30926235 PMC6477155

[51] Walker JA Clark PA Crisp A Barlow JL Szeto A Ferreira ACF Polychromic Reporter Mice Reveal Unappreciated Innate Lymphoid Cell Progenitor Heterogeneity and Elusive ILC3 Progenitors in Bone Marrow. Immunity (2019) 51(1):104–18.e7. 10.1016/j.immuni.2019.05.002 31128961 PMC6642165

[52] Cuff AO Sillito F Dertschnig S Hall A Luong TV Chakraverty R The Obese Liver Environment Mediates Conversion of NK Cells to a Less Cytotoxic ILC1-Like Phenotype. Front Immunol (2019) 10:2180. 10.3389/fimmu.2019.02180 31572388 PMC6749082

[53] Gordon SM Chaix J Rupp LJ Wu J Madera S Sun JC The transcription factors T-bet and Eomes control key checkpoints of natural killer cell maturation. Immunity (2012) 36(1):55–67. 10.1016/j.immuni.2011.11.016 22261438 PMC3381976

[54] Pikovskaya O Chaix J Rothman NJ Collins A Chen YH Scipioni AM Cutting Edge: Eomesodermin Is Sufficient To Direct Type 1 Innate Lymphocyte Development into the Conventional NK Lineage. J Immunol (2016) 196(4):1449–54. 10.4049/jimmunol.1502396 PMC4744497 26792802

[55] Park E Patel S Wang Q Andhey P Zaitsev K Porter S Toxoplasma gondii infection drives conversion of NK cells into ILC1-like cells. Elife (2019) 8. 10.7554/eLife.47605 PMC6703900 31393266

[56] Harmon C Robinson MW Fahey R Whelan S Houlihan DD Geoghegan J Tissue-resident Eomes(hi) T-bet(lo) CD56(bright) NK cells with reduced proinflammatory potential are enriched in the adult human liver. Eur J Immunol (2016) 46(9):2111–20. 10.1002/eji.201646559 27485474

[57] Stegmann KA Robertson F Hansi N Gill U Pallant C Christophides T CXCR6 marks a novel subset of T-bet(lo)Eomes(hi) natural killer cells residing in human liver. Sci Rep (2016) 6:26157. 10.1038/srep26157 27210614 PMC4876507

[58] Gao Y Souza-Fonseca-Guimaraes F Bald T Ng SS Young A Ngiow SF Tumor immunoevasion by the conversion of effector NK cells into type 1 innate lymphoid cells. Nat Immunol (2017) 18(9):1004–15. 10.1038/ni.3800 28759001

[59] Cortez VS Ulland TK Cervantes-Barragan L Bando JK Robinette ML Wang Q SMAD4 impedes the conversion of NK cells into ILC1-like cells by curtailing non-canonical TGF-beta signaling. Nat Immunol (2017) 18(9):995–1003. 10.1038/ni.3809 28759002 PMC5712491

[60] Wang S Xia P Chen Y Qu Y Xiong Z Ye B Regulatory Innate Lymphoid Cells Control Innate Intestinal Inflammation. Cell (2017) 171(1):201–16.e18. 10.1016/j.cell.2017.07.027 28844693

[61] Wang S Qu Y Xia P Chen Y Zhu X Zhang J Transdifferentiation of tumor infiltrating innate lymphoid cells during progression of colorectal cancer. Cell Res (2020). 10.1038/s41422-020-0312-y PMC7343789 32367039

[62] Morita H Kubo T Ruckert B Ravindran A Soyka MB Rinaldi AO Induction of human regulatory innate lymphoid cells from group 2 innate lymphoid cells by retinoic acid. J Allergy Clin Immunol (2019) 143(6):2190–201.e9. 10.1016/j.jaci.2018.12.1018 30682454

[63] Bando JK Gilfillan S Di Luccia B Fachi JL Secca C Cella M ILC2s are the predominant source of intestinal ILC-derived IL-10. J Exp Med (2020) 217(2). 10.1084/jem.20191520 PMC7041711 31699824

[64] Seehus CR Kadavallore A Torre B Yeckes AR Wang Y Tang J Alternative activation generates IL-10 producing type 2 innate lymphoid cells. Nat Commun (2017) 8(1):1900. 10.1038/s41467-017-02023-z 29196657 PMC5711851

[65] Geremia A Arancibia-Carcamo CV Innate Lymphoid Cells in Intestinal Inflammation. Front Immunol (2017) 8:1296. 10.3389/fimmu.2017.01296 29081776 PMC5645495

